# Polydatin Protects Rat Liver against Ethanol-Induced Injury: Involvement of CYP2E1/ROS/Nrf2 and TLR4/NF-κB p65 Pathway

**DOI:** 10.1155/2017/7953850

**Published:** 2017-11-08

**Authors:** Qiong-Hui Huang, Lie-Qiang Xu, Yu-Hong Liu, Jia-Zhen Wu, Xue Wu, Xiao-Ping Lai, Yu-Cui Li, Zi-Ren Su, Jian-Nan Chen, You-Liang Xie

**Affiliations:** ^1^Guangdong Provincial Key Laboratory of New Chinese Medicinal Development and Research, Mathematical Engineering Academy of Chinese Medicine, Guangzhou University of Chinese Medicine, Guangzhou 510006, China; ^2^The First Affiliated Hospital of Chinese Medicine, Guangzhou University of Chinese Medicine, 12 Airport Road, Baiyun District, Guangzhou 510405, China; ^3^Dongguan Mathematical Engineering Academy of Chinese Medicine, Guangzhou University of Chinese Medicine, Dongguan 523808, China; ^4^Higher Education Institute and Development Research of Chinese Medicine, 12 Airport Road, Baiyun District, Guangzhou 510405, China

## Abstract

Excessive alcohol consumption leads to serious liver injury, associating with oxidative stress and inflammatory response. Previous study has demonstrated that polydatin (PD) exerted antioxidant and anti-inflammatory effects and attenuated ethanol-induced liver damage, but the research remained insufficient. Hence, this experiment aimed to evaluate the hepatoprotective effect and potential mechanisms of PD on ethanol-induced hepatotoxicity. Our results showed that PD pretreatment dramatically decreased the levels of alanine aminotransferase (ALT), aspartate aminotransferase (AST), alkaline phosphatase (ALP), and lactate dehydrogenase (LDH) in the serum, suppressed the malonaldehyde (MDA) and triglyceride (TG) content and the production of reactive oxygen species (ROS), and enhanced the activities of superoxide dismutase (SOD), glutathione peroxidase (GSH-Px), catalase (CAT), andalcohol dehydrogenase (ADH), and aldehyde dehydrogenase (ALDH), paralleled by an improvement of histopathology alterations. The protective effect of PD against oxidative stress was probably associated with downregulation of cytochrome P450 2E1 (CYP2E1) and upregulation of nuclear factor erythroid 2-related factor 2 (Nrf2) and its target gene haem oxygenase-1 (HO-1). Moreover, PD inhibited the release of proinflammatory cytokines (TNF-*α*, IL-1*β*, and IL-6) via downregulating toll-like receptor 4 (TLR4) and nuclear factor kappa B (NF-*κ*B) p65. To conclude, PD pretreatment protects against ethanol-induced liver injury via suppressing oxidative stress and inflammation.

## 1. Introduction

Long-term excessive consumption of alcohol is harmful to the liver and inevitably induces alcoholic liver disease (ALD) [1]. The early stage of ALD is reflected as fatty liver, but it is finally progressing into more severe forms, such as inflammation, fibrosis, and cirrhosis [2]. Nowadays, ALD has become one of the leading causes of chronic diseases and death in the world [3]. Hence, it is necessary to unmask the pathological mechanisms of ALD and to look for potential therapeutic agents. It is well-known that liver is the major organ responsible for alcohol metabolism. In the liver, alcohol is converted to acetaldehyde mainly by alcohol dehydrogenase (ADH), cytochrome P450 2E1 (CYP2E1), and catalase and is further oxidized to acetate by aldehyde dehydrogenase (ALDH) and is finally converted to carbon dioxide via the citric acid cycle [4].

Many studies indicate that steatosis, oxidative stress, inflammatory factors, and mitochondrial dysfunction are involved in ALD [5]. And it is widely accepted that oxidative stress and inflammation are key mechanisms underlying the pathogenesis of ALD [3]. Reactive oxygen species (ROS), a highly reactive species of free radical, are known to play a dual role in living systems [6]. At physiological concentration, ROS play important roles in signal transduction, gene expression, and redox regulation. However, excessive production of ROS during pathological conditions has harmful effects, such as damage of DNA, proteins, and lipids [7]. Abundant evidences suggest that excessive alcohol consumption enhances the production of ROS [8]. And the main source of ROS in the liver is the cytochrome P45 enzymes [9, 10]. CYP 2E1 is of great importance in alcohol-induced generation of oxidative stress, not only for its activity enhancement induced by alcohol, but also for its participation in alcohol metabolism [9, 11]. Recently, it is reported that nuclear factor erythroid 2-related factor 2 (Nrf2) could protect against CYP2E1-induced oxidative stress through upregulation of antioxidant enzymes like HO-1 [12]. In addition, accumulating evidence has shown that chronic excessive alcohol intake also increases the level of inflammatory cytokines, such as TNF-*α*, IL-1*β*, and IL-6 [13]. The release of these cytokines is mainly associated with the activation of toll-like receptor 4 (TLR4) and its downstream nuclear factor kappa B (NF-*κ*B), which remains the key inflammatory pathway playing a vital role in alcohol-induced ALD model [14]. Hence, we deduce that antioxidant and anti-inflammatory therapy might improve the symptoms of ALD.

**Figure 1:**
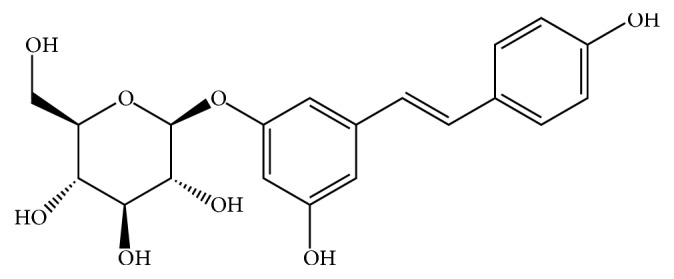
Chemical structure of polydatin.

Polydatin (PD), a glucoside of resveratrol (the structure was shown in Figure 1), known as a monocrystalline compound isolated from* Polygonum cuspidatum Sieb. et Zucc.*, is used for both medication and food [15]. PD is the richest active ingredient, whose concentration in* Polygonum cuspidatum Sieb. et Zucc.* is six times higher than resveratrol [16]. Besides, PD is also abound in the grape juices and exists widely in peanut, hop cones, cocoa-containing products, and chocolate products [17]. Mounting researches have focused on the biological activities of PD, such as antioxidant capacity, anti-inflammatory capacity, and multiple-organ protection. In our previous study, we found that PD attenuates D-galactose-induced liver injury [18]. Besides, PD has protective effect on CCl_4_-induced liver injury [15] and ethanol-induced liver injury in mice [19]. What is more, emerging evidence has demonstrated that resveratrol mitigates ethanol-induced oxidative stress in the liver of rats [20]. All these findings imply that PD is protective to the liver and might be a potential therapeutic agent for liver injury. The present study was aimed to explore the effect and the mechanism of PD on liver injury induced by alcohol using a rat ALD model.

## 2. Materials and Methods

### 2.1. Reagents

PD (purity above 98%) was supplied by Guangzhou Honsea Sunshine Biotech Co. Ethanol and silymarin were purchased from Sigma-Aldrich (purity above 98%, St Louis, Missouri, USA). Ethanol was diluted with distilled water to 56% (v/v). PD and silymarin were suspended in 0.5% carboxymethylcellulose (CMC-Na) distilled water solution. Triglyceride (TG) was purchased from Beijing BHKT Clinical Reagent Co. Ltd (Beijing, China). Primary antibodies against Nrf2, HO-1, CYP2E1, TLR4, NF-*κ*B p65, and *β*-actin and secondary antibodies were purchased from Santa Cruz Biotechnology (Santa Cruz, CA, USA). Other required materials are obtained from Sigma Co. LLC. (Guangzhou, China) with highest purity commercially available.

### 2.2. Animals and Experimental Protocols

Male Wistar rats (weighing 210 ± 10 g) were supplied by the Experimental Animal Center of Guangzhou University of Chinese Medicine. All procedures were performed in accordance with the Ethics Committee for the Welfare of Experimental Animals, of Guangzhou University of Chinese Medicine (No. 2017047). Before any experience, the animals were domesticated under the environment with the temperature of 25 ± 2°C, 50 ± 5% relative humidity, and a 12 : 12 h light-dark cycle and had free access to food and water. After one week of acclimatization, 72 rats were randomly assigned to six groups (twelve rats/group): normal control group, ethanol group, silymarin group (100 mg/kg) as the positive control, and three PD pretreatment groups (25, 50, and 100 mg/kg). Rats in Sily and PD groups were intragastrically given corresponding doses of drugs (10 mL/kg) once a day, while those in the NC and model groups received an equal volume of normal saline. The dose of PD was determined based on the previous investigation [21] and our preliminary experiments. After pretreatment for 7 consecutive days, all groups except NC group were given ethanol (7 ml/kg) intragastrically every 12 h at 5 different time points to establish the liver injury animal model. The ethanol dose was used according to a relative previous study [22], which was shown to induce severe hepatic damage and in which the administration times were confirmed by our preliminary tests. At the end of the experimental period (the 9th day), all the rats were anesthetized by intraperitoneal injection with pentobarbital (60 mg/kg) before being sacrificed. Blood samples were collected for serum biochemistry tests and the livers were quickly dissected, weighed, frozen in liquid nitrogen, and then stored at −80°C until analyzed.

### 2.3. Determination of Blood Biochemistry

The blood samples were placed at room temperature for 30 min and subsequently centrifuged at 3500 rpm for 15 min to separate the sera. Activities of alanine transaminase (ALT), aspartate amino transferase (AST), alkaline phosphatase (ALP), and lactate dehydrogenase (LDH) were measured using Hitachi - 7180 type biochemical analyzer following the standard protocol.

### 2.4. Histopathological Examination

Parts of the liver tissues were preserved in 10%  (v/v) phosphate buffered formalin (pH 7.4) for at least 24 h, dehydrated with a sequence of ethanol solutions (50–100%), embedded in paraffin wax, and sectioned for histopathological evaluation. Liver slices of 5-6 mm thickness were cut to stain with hematoxylin and eosin (H&E) while 10 *μ*m thick frozen ones were prepared to stain with Oil Red O solution according to standard protocols. Histopathologic changes could be examined and photographed at a magnification of ×200 with an Olympus IX71 microscope (Olympus Co., Tokyo, Japan).

### 2.5. Determination of Hepatic Triglyceride

Triglyceride (TG) levels, which is a marker of the liver steatosis, were analyzed by using a Triglyceride Quantification Kit following the manufacturer's instruction (Jiancheng Company, Nanjing, China). The results are expressed as mmol per mg protein (mmol/mg protein).

### 2.6. Measurement of Activities of Antioxidant Enzymes and ROS Production

Liver tissues were obtained to make 1 : 9 (w/v) homogenates with cold saline using a tissue tearor (T18 Basic, IKA). After centrifugation at 3500 rpm for 10 min at 4°C, the supernatant was collected to measure reactive oxygen species (ROS) production, superoxide dismutase (SOD), glutathione peroxidase (GSH-Px), and catalase (CAT) activities and malondialdehyde (MDA) content in liver by using commercial assay kits according to the manufacturer's protocols (Jiancheng Company, Nanjing, China).

### 2.7. Determination of Alcohol Dehydrogenase (ADH) and Aldehyde Dehydrogenase (ALDH) Levels

The ADH and ALDH levels in the liver homogenate were determined using commercial assay kits according to the manufacturer's protocols (Jiancheng Company, Nanjing, China).

### 2.8. Measurement of Inflammatory Cytokine Levels

Inflammatory cytokines including tumor necrosis factor (TNF-*α*), interleukin-1*β* (IL-1*β*), and interleukin-6 (IL-6) were measured using specific ELISA kits (eBioscience, USA).

### 2.9. Western Blotting Analyses

Total proteins were extracted from the liver tissues using protein extraction kit (Thermo) while the concentrations were measured by Protein Assay Kit (Nanjing, Jiancheng) according to the protocol. Equal amounts of protein (20 *μ*g) were separated on 10% SDS-polyacrylamide gel and transferred to 0.45 *μ*m polyvinylidene fluoride (PVDF) membranes. Then the membranes were blocked for 1 h at room temperature with Tris buffered saline (TBS) with 5% fat-free dry milk, followed by incubating with primary antibody of 1 : 500 Nrf2, 1 : 1000 HO-1, 1 : 1000 CYP2E1, 1 : 1000 TLR4, 1 : 1000 NF-*κ*B p65, and 1 : 1000 *β*-actin overnight at 4°C. Subsequently, the membranes were incubated with HRP-conjugated secondary antibody. Blots were then developed with the ECL reagent and were exposed by Western Blotting Detection System (Amersham Life Science, UK). The level of each band was subsequently quantitated by densitometry analysis using Image J, version 1.47v, software.

### 2.10. Statistical Analysis

The recorded parameters were expressed as mean ± standard deviation (mean ± SD) for all groups. One-way analysis of variance (one-way ANOVA) followed by post hoc and least significant difference (LSD) tests was used to statistically analyze the data by SPSS (version 16.0) software. *p* < 0.05 was considered to indicate statistically significant differences.

## 3. Results

### 3.1. PD Alleviated Abnormal Increase of Liver Functional Markers Induced by Alcohol

**Figure 2:**
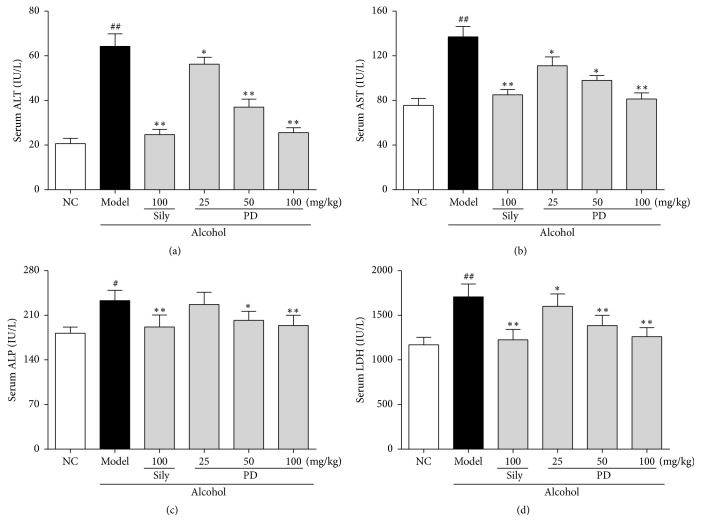
Effect of PD treatment on hepatic function (*n* = 12) in rats. The serum levels of (a) ALT, (b) AST, (c) ALP, and (d) LDH were determined. Values were presented as the mean ± SD. ^#^*p* < 0.05, ^##^*p* < 0.01 versus NC group; ^**∗**^*p* < 0.05, ^**∗∗**^*p* < 0.01 versus model group.

Hepatic function is always assessed by serum levels of ALT, AST, ALP, and LDH. Increased release of these hepatic functional markers reflects liver injury. As shown in Figures 2(a)–2(d), serum levels of ALT, AST, ALP, and LDH in the model group were increased by 206.4% (20.7 ± 2.4 versus 63.3 ± 6.0), 74.3% (78.1 ± 6.4 versus 136.1 ± 7.1), 29.0% (180.3 ± 8.0 versus 232.4 ± 10.3), and 45.7% (1168.9 ± 65.2 versus 1703.4 ± 65.9), respectively, as compared to the NC group (*p* < 0.01 or *p* < 0.05). In contrast, rats in PD-treated groups remarkably reversed the increase of these typical markers in a dose-dependent manner. Administration with PD at 100 mg/kg showed the best protective effect, which was comparable to that of the positive control silymarin (100 mg/kg).

### 3.2. PD Improved Ethanol-Induced Live Pathological Changes

**Figure 3:**
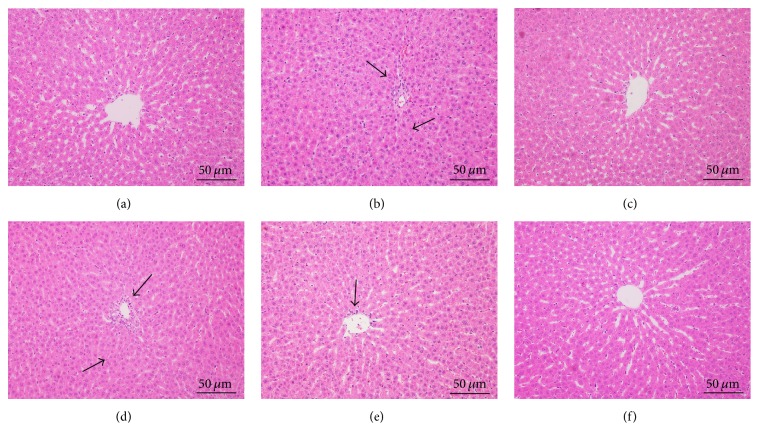
Effect of PD treatment on liver histological changes by H&E staining (200x). Representative histopathological alterations of liver are displayed of different groups. (a) Normal control group; (b) Ethanol model group; (c) Ethanol + Silymarin 100 mg/kg; (d) Ethanol + PD 25 mg/kg; (e) Ethanol + PD 50 mg/kg; (f) Ethanol + PD 100 mg/kg. Arrows indicated both apparent focal necrosis and inflammatory infiltration. Obviously normal morphology and great improvement in hepatocyte structure with silymarin and high-dose PD, similar to that of NC group.

Hepatic histological changes can directly reflect the degree of liver injury and repairment. To morphologically characterize the potential pathological alternations in the liver, H&E staining was performed. As shown in Figure 3(a), clear structure and regular lobular were observed, where hepatic cords remained legible and radiated from central vain in NC group. Compared to the NC group, loss of cellular boundaries, apparent hepatic parenchymal necrosis, inflammatory cell infiltration, and disordered hepatic cords accompanied by extensive vacuolation congestion were observed in model group (Figure 3(b)). Additionally, hepatocytes were extremely swollen, with condensed karyon and loosened cytoplasts. Following the silymarin or PD treatments, the impaired cells were progressively recovered, inflammatory reaction were significantly inhibited, and necrotic and apoptotic mass were reduced (Figures 3(c)–3(f)). PD of high dose showed the best effect on modulating these histological alterations and restoring disorder of hepatic arrangement.

### 3.3. PD Suppressed Ethanol-Induced Hepatic Steatosis

**Figure 4:**
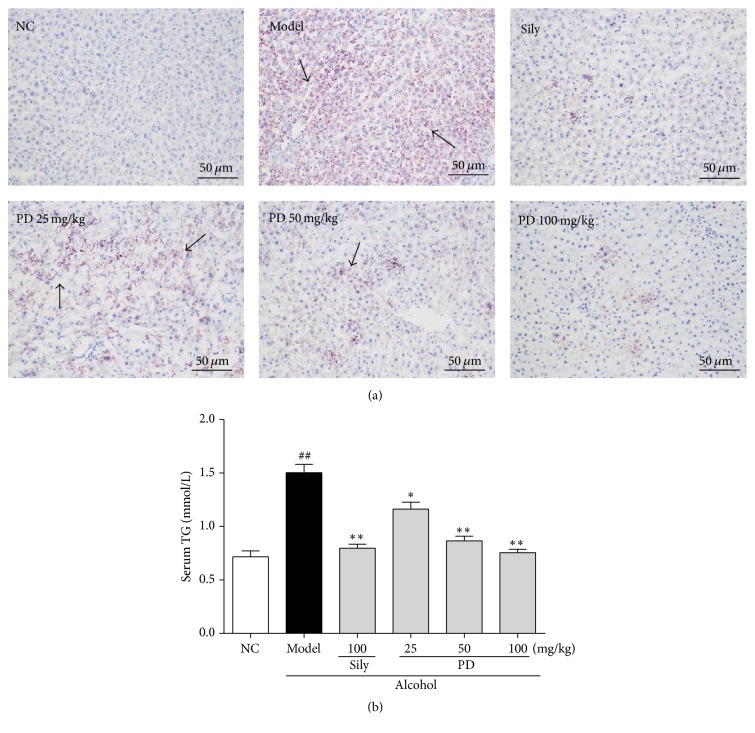
Effect of PD on hepatic lipid accumulation in liver tissues. Representative of (a) Oil Red O-stained photos from each group and (b) TG level are presented. NC: normal control group; Model: ethanol model group; Sily: Ethanol + Silymarin 100 mg/kg; PD 25 mg/kg: Ethanol + PD 25 mg/kg; PD 50 mg/kg: Ethanol + PD 50 mg/kg; PD 100 mg/kg: Ethanol + PD 100 mg/kg. Arrows indicated appearance of prominent microvesicular steatosis. Values were presented as the mean ± SD. ^##^*p* < 0.01 versus NC group; ^**∗**^*p* < 0.05, ^**∗∗**^*p* < 0.01 versus model group.

In order to assess the effect of PD on hepatic lipid accumulation induced by ethanol metabolism, Oil Red O staining was examined. Obvious microvesicular steatosis and large amount of lipid droplets were visualized in the livers of model group, as compared to NC group (Figure 4(a)). Interestingly, PD and silymarin treatment remarkably decreased the amount of hepatic lipid droplets. Lipid accumulation in the liver was further confirmed by quantitative analysis of hepatic TG content (Figure 4(b)). The results showed that TG level was dramatically increased in model group by 108.3% in comparison to control group (*p* < 0.05), but this elevation was apparently diminished by either silymarin or PD treatment. It was worth noting that PD at the dose of 100 mg/kg could even restore the levels of serum lipids to normal. These data clearly manifest that PD could effectively protect the liver against ethanol-induced steatosis.

### 3.4. PD Augmented Activities of Ethanol Metabolic Enzymes ADH and ALDH

**Figure 5:**
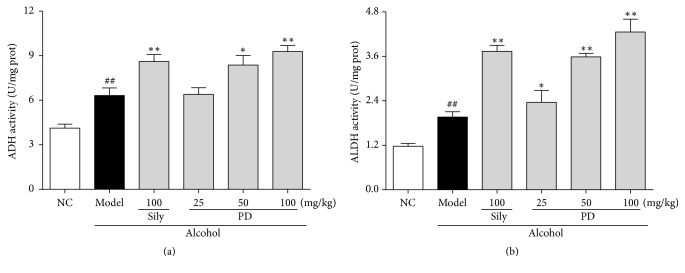
Effect of PD on ethanol metabolic enzymes activities of (a) ADH and (b) ALDH. Values were presented as the mean ± SD. ^##^*p* < 0.01 versus NC group; ^**∗**^*p* < 0.05, ^**∗∗**^*p* < 0.01 versus model group.

ADH and ALDH are two key enzymes participating in alcohol metabolism. Our results showed that hepatic ADH and ALDH activity were significantly increased in model group by 53.0% and 67.5% (Figure 5), respectively, as compared with those in the NC group (*p* < 0.05). Interestingly, the activities of these enzymes were further elevated by PD administration, suggesting that pretreatment with PD prevents ethanol-induced liver injury partly through promoting ethanol metabolism by augmenting the activities of ADH and ALDH.

### 3.5. PD Ameliorated Ethanol-Induced Oxidative Stress and Lipid Peroxidation in Liver

**Figure 6:**
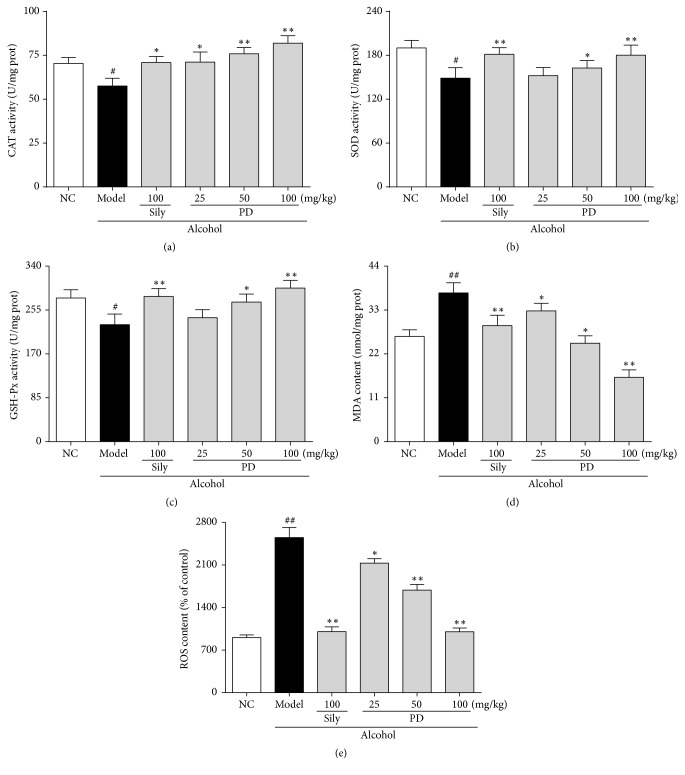
Effect of PD on activities of antioxidant enzymes and level of MDA and ROS in liver. (a) CAT; (b) SOD; (c) GSH-Px; (d) MDA; (e) ROS. Values were presented as the mean ± SD. ^#^*p* < 0.05, ^##^*p* < 0.01 versus NC group; ^**∗**^*p* < 0.05, ^**∗∗**^*p* < 0.01 versus model group.

Oxidative stress and lipid peroxidation caused by alcohol accumulation play a critical role in liver injury. To evaluate the hepatoprotective effects of PD on oxidative stress, the activities of hepatic antioxidant enzymes such as SOD, GSH-Px, and CAT were determined. As shown in Figures 6(a)–6(c), the activities of antioxidant enzymes (CAT, SOD, and GSH-Px) were conspicuously reduced in the model group to 78.3%, 79.3%, and 82.8%, respectively, as compared with those in the NC group (*p* < 0.05). On the contrary, administration with silymarin (100 mg/kg) and PD (50, 100 mg/kg), significantly restored these enzyme activities in the liver. Furthermore, 100 mg/kg of PD dramatically elevated the activities of CAT and GSH-Px, even surpassing the normal level. MDA level and ROS production, which are important indicators of lipid peroxidation, were detected and shown in Figures 6(d) and 6(e). Acute ethanol exposure induced excessive ROS production and MDA level in liver, which were markedly attenuated by either silymarin or high-dose PD treatment. Briefly, in comparison with the model group, ROS production was decreased by 60.7%  (*p* < 0.01) and MDA level reduced by 56.2%  (*p* < 0.01) in high-dose PD (100 mg/kg). The effect of PD at high dose was comparable to that of the silymarin group.

### 3.6. PD Modulated the Expressions of CYP2E1, Nrf2, and OH-1 in Liver

**Figure 7:**
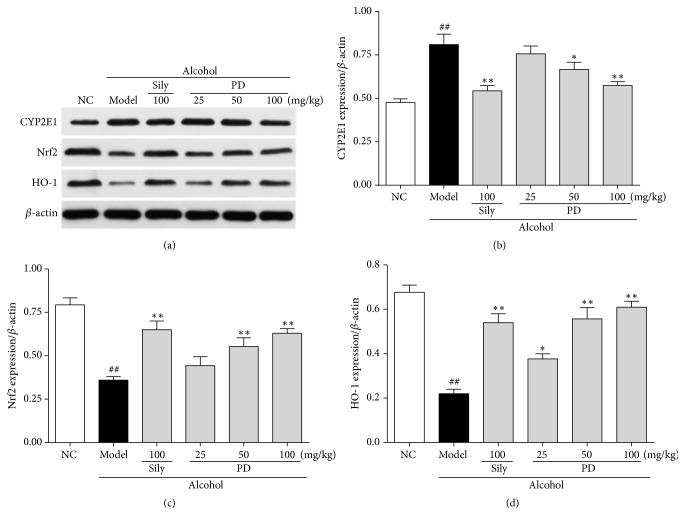
Effect of PD on ethanol-induced oxidative stress. (a) Western blot analysis of CYP2E1, Nrf2, and HO-1 proteins. (b) Quantitative analysis of CYP2E1/*β*-actin ratio. (c) Quantitative analysis of Nrf2/*β*-actin ratio. (d) Quantitative analysis of HO-1/*β*-actin ratio. Values were presented as the mean ± SD. ^##^*p* < 0.01 versus NC group; ^**∗**^*p* < 0.05, ^**∗∗**^*p* < 0.01 versus model group.

To understand the mechanism underlying the protective effect of PD on ethanol-induced oxidative stress, the protein expressions of CYP2E1, Nrf2, and HO-1 were measured by immunoblotting analysis. As shown in Figures 7(a) and 7(b), the expression of CYP2E1 in ethanol group was significantly increased by 1.7-fold when compared with the control group. This elevation was dramatically prevented by PD at the dose of 50 mg/kg and 100 mg/kg. Conversely, in comparison with normal control group, the expressions of Nrf2 and its downstream target protein HO-1 in the model group were decreased by 46% and 32% (Figures 7(a), 7(c), and 7(d)), respectively. However, the decrease was reversed by the treatments of PD and silymarin. Our data demonstrate that PD ameliorates oxidative stress via upregulation of Nrf2 and HO-1 and downregulation of CYP2E1.

### 3.7. PD Inhibited Ethanol-Induced Hepatic Inflammatory Response

**Figure 8:**
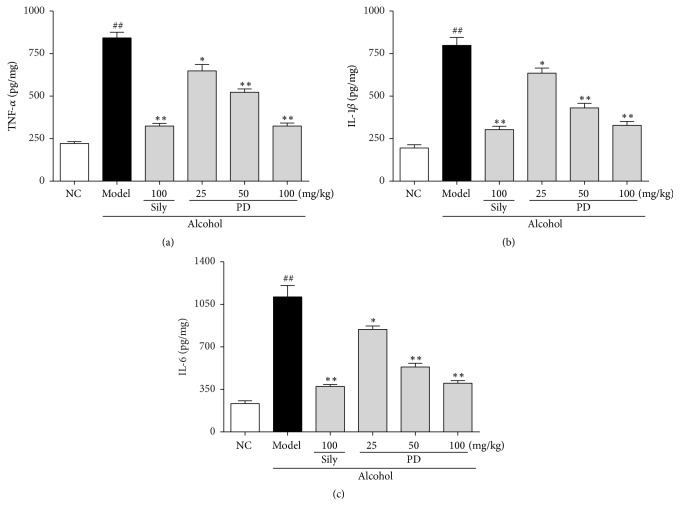
Effect of PD on inflammatory cytokines of TNF-*α*, IL-1*β*, and IL-6 in liver. (a) TNF-*α*, (b) IL-1*β*, and (c) IL-6. Values were presented as the mean ± SD. ^##^*p* < 0.01 versus NC group; ^**∗**^*p* < 0.05, ^**∗∗**^*p* < 0.01 versus model group.

Excessive inflammatory response could activate stress signal and induce oxidative stress [23]. In the present study, the levels of several important inflammatory cytokines such as TNF-*α*, IL-1*β*, and IL-6 were determined. The levels of these cytokines were significantly higher in the model group than those in the NC group (Figure 8), indicating that ethanol-induced inflammatory response in the liver. Promisingly, pretreatment with PD inhibited the productions of these proinflammatory factors in a dose-dependent manner (*p* < 0.01). It is suggested that PD might attenuate alcohol-induced liver injury via suppressing the inflammatory response.

### 3.8. PD Attenuated the Expressions of TLR4 and p65 in the Liver

**Figure 9:**
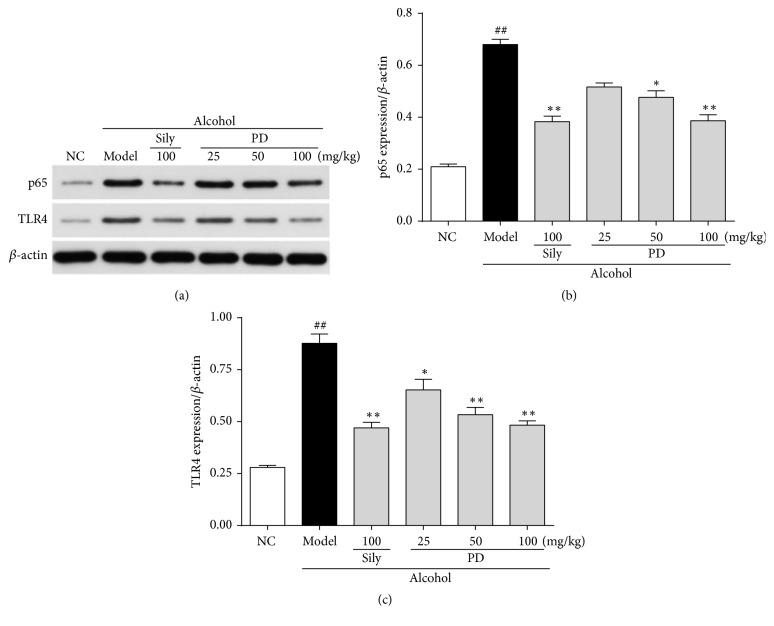
Effect of PD on ethanol-induced inflammatory response. (a) Western blot analysis of p65 and TLR4 proteins. (b) Quantitative analysis of p65/*β*-actin ratio. (c) Quantitative analysis of TLR4/*β*-actin ratio. Values were presented as the mean ± SD. ^##^*p* < 0.01 versus NC group; ^**∗**^*p* < 0.05, ^**∗∗**^*p* < 0.01 versus model group.

To explore whether or not the classical inflammatory signaling pathway TLR4-NF-*κ*B is involved in the anti-inflammatory effect of PD, the expressions of TLR4 and NF-*κ*B subunit p65 were analyzed. The protein expressions of TLR4 and its downstream NF-*κ*B p65 in the model group were enhanced by 3.1-fold and 3.2-fold, respectively, which were reversed by silymarin and PD groups (Figure 9). Herein, these observations indicate that the anti-inflammatory effect of PD on alcohol-induced acute liver injury is via suppressing TLR4-NF-*κ*Bp65 pathway.

## 4. Discussions

Alcoholic beverages consumption has a long history and now it has been a part of human diet throughout the word. However, lots of health problems, diseases, and even deaths are attributable to excessive consumption of alcohol. Heretofore, the precise mechanisms of ALD still remain unclear, and effective drugs need developing urgently. According to a previous study [3], ameliorating fatty degeneration, suppressing oxidative stress, and inhibiting inflammation have been considered to be promising therapeutic strategies for ALD. Therefore, our research was aimed to seek out potential therapeutic agents against ethanol-induced hepatotoxicity based on these aspects. Herbal drugs play a crucial role in the regeneration of liver cells, acceleration of healing process, and hence management of various liver disorders [24]. As a common constituent of natural products, PD has been found in many foods like grapes and peanuts, so it is considered to be safe for adding into daily meals as food supplement for treating diseases so as to expand its application. To date, PD has caught great attention for its remarkable curative effect on both D-galactose- [18] and CCl_4_- [15] induced liver injury. Besides, PD has also been found to have a benefit in alleviating ethanol-induced liver damage by refurbishing the matrix metalloproteinases levels and attenuating oxidative stress in mice [19]. In our experiments, we provide additional evidence that PD pretreatment was able to exert protective effect against ethanol-induced hepatotoxicity by elevating antioxidant capacity, preventing inflammatory response and the relevant signaling pathways, and ameliorating the steatosis of liver cells.

Liver injury is commonly evaluated by serum biochemical markers and histological analyses. Serum AST, ALT, ALP, and LDH are among the most sensitive biochemical markers employed in the assessment of liver function. It is proved that AST, AST, ALP, and LDH can be expressed in the liver and that abnormal upregulation of these enzymes would cause damage and necrosis of hepatic cells [25, 26]. When the permeability of hepatocellular membrane is enhanced under pathological conditions, these cellular enzymes are released into the bloodstream, resulting in an elevation of serum enzyme levels. Previous studies have found that oral administration of alcohol to rats could induce acute hepatic injury and obviously elevate levels of ALT, AST, ALP, and ADH in the serum [27]. In the present work, administration of ethanol to rats in the model group led to liver damage as evidenced by an obvious elevation in serum AST, ALT, ALP, and LDH, indicating that the pathological condition of severe liver injury is congruent with previous reports [28, 29]. Notably, PD pretreatment was shown to markedly reduce the increased levels of these markers, which was indicative of improvement of hepatic function. In accordance with the alteration of liver function markers, this biochemical analysis was further validated by histopathological observations. Alcohol caused obvious damage to the hepatic cell structure and produced serious pathological alternations including vacuole formation, inflammation infiltration, and focal necrosis. However, pretreatment with PD significantly improved these deteriorations of hepatic cells. Hepatoprotection of PD was particularly evident from the inhibition of inflammatory reactions and cellular lipoidosis in the liver section of rats treated with middle and high doses.

ALD is a progressive disease, which might progress to alcoholic steatohepatitis, and is incurable in an advanced stage. In the normal rats, the liver could maintain a stable state of equilibrium of lipid metabolism, which could be broken when the liver is subjected to stimulation of excessive alcohol and therefore leading to fatty degeneration and alcoholic liver injury [30]. Large amounts of alcohol consumption can damage hepatocyte mitochondrial function, inhibit peroxidase activity, and further interfere in lipid oxidation reaction, ultimately resulting in lipid accumulation [31, 32]. Moreover, alcohol can also increase mobilization of fatty acids in the liver by stimulating peripheral steatolysis and promote TG synthesis [33]. In line with these findings, our observations suggest that lipid metabolic disorder is induced after alcohol administration, as implied by the increase of TG content and the results of Oil Red O staining. Oil Red O is a fat-soluble dye that can stain fats and triglycerides contained in the injured liver and thus it is used for assessing the extent of ethanol-induced lipid degeneration. Alcohol led to structure loosening, fat deposits, and lesions in wide distribution of liver. Previous studies have indicated that PD exerts lipid-lowering effects in liver disease model by decreasing the production of TG [16, 34]. According to our observations, PD treatment effectively improved hepatic steatosis by not only decreasing TG content, but also suppressing lipid deposition shown by Oil Red O staining. Thus, these results suggest that PD significantly prevents the development of fatty liver and helps to repair hepatocytes especially at high doses.

The present study also demonstrated that PD enhanced ethanol metabolism to protect the liver against ethanol-induced injury. The metabolism of ethanol contains two steps, of which firstly ethanol is converted to acetaldehyde primarily by ADH, and acetaldehyde is further metabolized to acetate by ALDH as the second step. Acetaldehyde is a toxic intermediate through its interactions with proteins and lipids which can lead to free radical formation and cell damage [35]. As a compensatory mechanism, the activities of both ADH and ALDH will be increased to remove the toxic alcohol and acetaldehyde when ethanol intake is excessive [36, 37]. According to our results, hepatic ADH and ALDH activities were significantly elevated in alcohol model group. Surprisingly, PD administration further augmented the activities of these two enzymes as compared with ethanol group. The enhancement of this compensatory mechanism by PD might contribute to its hepatoprotective effects by stimulating ethanol metabolism [26, 38]. Additionally, our results also demonstrated that the increase of ALDH level was much more potent than that of the ADH level, suggesting that PD is able to avoid acetaldehyde accumulation. Therefore, the effects of PD on elevating ADH and ALDH activities might at least partially contribute to its hepatoprotective effects.

It has been well accepted that oxidative stress, caused by an imbalance in prooxidants and antioxidants [39], is involved in molecular mechanism underlying the pathogenesis of ethanol-induced hepatic damage [40]. During the activation of ADH and ALDH by alcohol intake, CYP2E1, the major hepatic metabolic enzyme in liver microsomes, accelerates the metabolism of alcohol and formation of acetaldehyde to promote ROS generation [5, 16, 35–37]. ROS reacts with most biological macromolecules, such as DNA, proteins, and polyunsaturated fatty acids on the cell membrane, causing lipid peroxidation (LPO) and subsequently developing hepatotoxicity [41]. MDA, as the end-product of LPO, is a typical indicator to evaluate the degree of oxidative stress [42]. It has been reported that CYP2E1 knockout notably suppresses the increase of ROS and MDA to alleviate alcohol-induced injury [43]. As shown in our study, PD lowered the protein expression of CYP2E1 as well as the overproduction of ROS and MDA, consistent with the previous findings. It is possible that the liver protective effect of PD against ethanol might be attributed to counteracting oxidative stress via inhibition of CYP2E1. SOD and GSH-Px are important nonenzymatic antioxidants to keep cellular redox balance, not only scavenging oxygen free radicals but also decreasing lipid peroxidation in tissues [3]. It has been recognized that upregulation of SOD with adenovirus infection could obviously reduce ROS level and strengthen liver regeneration and function in response to alcohol [44]. The present work revealed that pretreatment with PD could rebound the reduction of the activities of antioxidant enzymes (SOD and GSG-Px), to nearly normal level in high-dose treatment group. Moreover, we investigated the protein expressions of Nrf-2 and HO-1 in order to find out the mechanisms underlying the dramatic enhancement of oxidant defense systems by PD treatment. Nrf-2, a transcription factor that induces a variety of downstream genes encoding detoxification enzymes and antioxidant proteins in response to oxidative stress [5], has recently been identified as a novel therapeutic target for ALD [45]. HO-1, the downstream protein of Nrf-2, plays a key role in strengthening resistance capacity and restraining redox disorder when liver is attacked by alcohol. A mass of correlative literatures has proved that the CYP2E1/ROS/Nrf2 signaling pathway is involved in alcohol-induced hepatic injury [46–50]. Our findings manifested that PD pretreatment not only reversed the upregulation of CYP2E1 and the increase of ROS and MDA levels, but significantly enhanced the decreased expression of hepatic Nrf2 and HO-1 induced by alcohol. Taken together, protective effect of PD against ethanol-induced livery injury might be attributed to suppressing oxidative stress through restoring antioxidant system via the activation of CYP2E1/ROS/Nrf2 pathway.

Inflammation is another representative pathological mechanism that is responsible for ethanol-elicited liver damage. Oxidative stress can trigger expression of proinflammatory proteins to facilitate inflammatory responses, which aggravates liver injury [51]. It has been reported that large amount of alcohol intake activates the TLR4/NF-*κ*B signaling pathway due to overproduction of ROS [3, 52, 53]. TLR4, a cytomembrane TLR, acts as an indispensable role in the pathological progression of inflammatory liver diseases [54]. TLR4 can identify extraneous molecular patterns which are associated with liver damage [55, 56]. Activation of the TLR4 leads to translocation of NF-*κ*B p65 from cytoplasm to the nucleus. Known as downstream effector proteins of TLR4, NF-*κ*B p65 participates in gene transcription of cytokines, growth factors, and oxidative stress-related enzymes, to further regulate physiologic or pathologic events such as inflammatory and immune response. When stimulated, NF-*κ*B p65 will bind to DNA and trigger the production of inflammatory mediators, such as TNF-*α*, IL-1*β*, and IL-6. Accumulation of TNF-*α*, IL-1*β*, and IL-6 are thought to be the key factors in the pathogenesis of ALD because of the disruption of the balance between proinflammation and anti-inflammation [57–60]. Therefore, suppression of the inflammatory reaction is a crucial strategy to ameliorate ethanol-induced liver injury. It has been suggested that resveratrol, which shared similar chemical structure with PD, possessed satisfactory anti-inflammatory effect against liver damage [61–63]. Therefore, we hypothesized that PD might have similar effect on ethanol-induced inflammation. In our experiments, we found that ethanol exposure obviously increased protein expressions of TLR4 and NF-*κ*B p65 and subsequently elevated TNF-*α*, IL-1*β*, and IL-6 levels. As expected, PD pretreatment could apparently limit TLR4 and NF-*κ*B p65 expression and thus suppress the formation of inflammatory cytokines (TNF-*α*, IL-1*β*, and IL-6), suggesting that PD provides anti-inflammatory effect against ethanol-induced liver injury via suppressing the TLR4/NF-*κ*B p65 signal pathway.

Interestingly, the interplay between oxidative stress and inflammation in ethanol-induced liver damage has been discussed by multiple studies [64–66]. Oxidative stress can trigger the expressions of proinflammatory proteins to facilitate inflammatory responses, of which NF-*κ*B activation is involved [67–72]. On the other hand, NF-*κ*B, as a transcriptional factor participating in acute inflammatory reactions, is associated with increased generation of ROS [73–76]. Our observations that PD suppresses oxidative stress (probably via the CYP2E1/ROS/Nrf2 pathway) and inflammatory response (probably via TLR4/NF-*κ*B p65 signal pathway) might hint that PD participates in the regulation of interplay between oxidative stress and inflammation. Further studies will be required to unveil the mechanisms underlying the regulation of PD on the interplay of these signaling pathways.

## 5. Conclusion

Short-term oral administration of PD has been proved to exert a pronounced effect on elevating antioxidant enzymes activities to relieve ethanol-induced oxidative stress and inhibiting proinflammatory cytokines expressions to mitigate liver impairment, by which the regulation of CYP2E1/ROS/Nrf2 and TLR4/NF-*κ*B pathway might probably be involved. These findings provide solid evidence that PD could protect against ethanol-induced liver damage.
